# Draft Genome Sequence of Microcystis aeruginosa NIES-4285, Isolated from Brackish Water (Lake Abashiri, Japan)

**DOI:** 10.1128/MRA.00001-19

**Published:** 2019-02-07

**Authors:** Yuuhiko Tanabe, Haruyo Yamaguchi

**Affiliations:** a Algae Biomass and Energy System R&D Center, University of Tsukuba, Ibaraki, Japan; b Center for Environmental Biology and Ecosystem Studies, National Institute for Environmental Studies, Ibaraki, Japan; University of Maryland School of Medicine

## Abstract

Microcystis aeruginosa is a bloom-forming cyanobacterium found in fresh and brackish waters worldwide. We sequenced the whole genome of M. aeruginosa NIES-4285, isolated from Lake Abashiri, Japan. Its genome contains approximately 5.2 Mbp with an average G+C content of 42.60% and is predicted to have 4,980 protein-coding genes.

## Abstract (precis)

Microcystis aeruginosa is a bloom-forming cyanobacterium found in fresh and brackish waters worldwide. We sequenced the whole genome of M. aeruginosa NIES-4285, isolated from Lake Abashiri, Japan.

## ANNOUNCEMENT

Microcystis aeruginosa is a bloom-forming cyanobacterium that is found in fresh and brackish waters worldwide (1). Currently, >35 whole-genome sequences of M. aeruginosa are available in GenBank (2). However, most of these strains are of freshwater origin; only three sets of whole-genome data of brackish water strains have been reported (3). To gain insight into the genomic basis of the occurrence of M. aeruginosa blooms in brackish water, we sequenced the whole genome of M. aeruginosa NIES-4285, which had recently been isolated from a Japanese lake with brackish water, Lake Abashiri.

The axenic strain M. aeruginosa NIES-4285 was isolated from a bloom collected from Lake Abashiri, Japan; salinity at the time of sampling was 0.6 practical salinity units (PSU). The strain was cultured in 100 ml of MA medium (4) in a 200-ml Erlenmeyer flask for 4 weeks. Cells were harvested by centrifugation (20,000 × *g*), and genomic DNA was extracted using NucleoBond AXG columns with buffer set III (Macherey-Nagel, Düren, Germany). DNA was sheared into approximately 450 bp fragments using a Covaris M220 ultrasonicator (Covaris, Woburn, MA). A 450-bp fragment library was constructed using the NEBNext Ultra DNA library prep kit for Illumina (New England Biolabs, Ipswich, MA). The DNA sequencing was performed with the MiSeq platform (Illumina, San Diego, CA) using the 500-cycle MiSeq reagent kit v2, which resulted in 1,274,970 paired-end reads. Low-quality reads/bases were filtered using Trimmomatic v0.36 (5), and *de novo* assembly was performed with SPAdes v3.10.1 (6). The genome was assembled into 444 contigs of 5,254,657 bp with an *N*_50_ value of 38,295 bp. The average genome coverage of the paired-end reads was 86×, and the maximum contig length was 159,106 bp. After removing contaminants and short contigs (<200 bp), the draft genome sequence of M. aeruginosa NIES-4285 was annotated using Prokka v1.12 (7). The genome contained 4,980 predicted protein-coding sequences (CDSs), including 2,803 hypothetical proteins and 52 RNA genes. The G+C content of the genome was 42.60%. Unlike three known salt-tolerant M. aeruginosa strains isolated from brackish water (3), M. aeruginosa NIES-4285 does not possess genes for osmoprotectant sucrose (3, 8). Furthermore, genes for other osmoprotectants, such as trehalose and glucosylglycerol (8), were not detected. This finding is consistent with the observation that NIES-4285 could not grow in culture at salinity of >5 PSU. M. aeruginosa is a rich source of various bioactive secondary metabolites, including hepatotoxic polypeptide microcystins (9). Genes for microcystin biosynthesis (*mcy*) (9) were not detected; in contrast, genes for cyanopeptolin (*mcn*) (10) and aeruginosin (*aer*) (11) biosynthesis were found in the NIES-4285 genome. This draft genome sequence provides genetic information for future investigation of the genomic diversity and bloom occurrence of M. aeruginosa in brackish water.

### Data availability.

This whole-genome shotgun project has been deposited in DDBJ under the accession no. BIFY01000000. Raw sequencing reads have been deposited in DDBJ under the accession no. DRR164041.
